# Integrated Assessment of Genomic Correlates of Protein Evolutionary Rate

**DOI:** 10.1371/journal.pcbi.1000413

**Published:** 2009-06-12

**Authors:** Yu Xia, Eric A. Franzosa, Mark B. Gerstein

**Affiliations:** 1 Bioinformatics Program, Boston University, Boston, Massachusetts, United States of America; 2 Department of Chemistry, Boston University, Boston, Massachusetts, United States of America; 3 Department of Molecular Biophysics and Biochemistry, Yale University, New Haven, Connecticut, United States of America; Stanford University, United States of America

## Abstract

Rates of evolution differ widely among proteins, but the causes and consequences of such differences remain under debate. With the advent of high-throughput functional genomics, it is now possible to rigorously assess the genomic correlates of protein evolutionary rate. However, dissecting the correlations among evolutionary rate and these genomic features remains a major challenge. Here, we use an integrated probabilistic modeling approach to study genomic correlates of protein evolutionary rate in *Saccharomyces cerevisiae*. We measure and rank degrees of association between (i) an approximate measure of protein evolutionary rate with high genome coverage, and (ii) a diverse list of protein properties (sequence, structural, functional, network, and phenotypic). We observe, among many statistically significant correlations, that slowly evolving proteins tend to be regulated by more transcription factors, deficient in predicted structural disorder, involved in characteristic biological functions (such as translation), biased in amino acid composition, and are generally more abundant, more essential, and enriched for interaction partners. Many of these results are in agreement with recent studies. In addition, we assess information contribution of different subsets of these protein properties in the task of predicting slowly evolving proteins. We employ a logistic regression model on binned data that is able to account for intercorrelation, non-linearity, and heterogeneity within features. Our model considers features both individually and in natural ensembles (“meta-features”) in order to assess joint information contribution and degree of contribution independence. Meta-features based on protein abundance and amino acid composition make strong, partially independent contributions to the task of predicting slowly evolving proteins; other meta-features make additional minor contributions. The combination of all meta-features yields predictions comparable to those based on paired species comparisons, and approaching the predictive limit of optimal lineage-insensitive features. Our integrated assessment framework can be readily extended to other correlational analyses at the genome scale.

## Author Summary

Proteins encoded within a given genome are known to evolve at drastically different rates. Through recent large-scale studies, researchers have measured a wide variety of properties for all proteins in yeast. We are interested to know how these properties relate to one another and to what extent they explain evolutionary rate variation. Protein properties are a heterogeneous mix, a factor which complicates research in this area. For example, some properties (e.g., protein abundance) are numerical, while others (e.g., protein function) are descriptive; protein properties may also suffer from noise and hidden redundancies. We have addressed these issues within a flexible and robust statistical framework. We first ranked a large list of protein properties by the strength of their relationships with evolutionary rate; this confirms many known evolutionary relationships and also highlights several new ones. Similar protein properties were then grouped and applied to predict slowly evolving proteins. Some of these groups were as effective as paired species comparison in making correct predictions, although in both cases a great deal of evolutionary rate variation remained to be explained. Our work has helped to refine the set of protein properties that researchers should consider as they investigate the mechanisms underlying protein evolution.

## Introduction

Different proteins evolve at drastically different rates [1]. Some proteins are highly conserved across distantly diverged species, such as the ribosomal and histone proteins in eukaryotes [2]. Other proteins evolve much more quickly, often to the point where they occur in one species but cannot be identified in other closely related species, possibly due to deletion or major sequence divergence [3]. What are the main driving forces of such differences in protein evolutionary rate? What percentage of this variation can be attributed to simple protein properties that we can quantitatively measure in a genome-wide fashion? The answers to such questions are critical to achieving a systematic understanding of molecular evolution.

With the advent of reliable high-throughput functional genomic measurements, particularly in the model organism *Saccharomyces cerevisiae* (baker's yeast), it is now possible to rigorously assess the functional genomic correlates of protein evolutionary rate. Many studies have focused on calculating the correlation between protein evolutionary rate and a single protein feature that can be determined for a large fraction of yeast proteins, followed by statistical hypothesis testing of the observed correlation. This method has been successful in identifying a number of key correlates of protein evolutionary rate, such as protein abundance [4], essentiality [5], and number of interactors [6]. For further review of individual correlates, see [1],[7],[8].

Assessing the relative strengths, synergistic effects, and redundancy among such correlations requires more sophisticated statistical methods. Multivariate techniques have already been applied in a number of studies aimed at simultaneously dissecting multiple correlates of evolutionary rate [9]–[16]. Partial correlation and principle component regression, two popular techniques in this area, have been shown to produce discrepant results when applied to similar data [13],[15],[17]. Arguments have been made against both techniques regarding their sensitivity to noise among protein features and a tendency to over- or under-estimate the number of independent determinants of evolutionary rate.

Analyses of evolutionary rate correlation have been historically limited by less-than-complete coverage of the genome—often far less. Consider the calculation of evolutionary rate itself. A commonly used reference dataset was produced by Wall et al., where a set of evolutionary rate calculations were meticulously performed in yeast [12]. In generating these data, they placed demands on gene orthology and phylogenetic relationships that substantially reduced genome coverage (to roughly 3,000 genes, relative to the roughly 6,000 open reading frames in *S. cerevisiae*). Moreover, a reduction in genome coverage may be accompanied by the introduction of specific biases. For example, stringent demands on gene orthology automatically bias a dataset toward more slowly evolving proteins. Coverage will tend to be further limited—and the dataset further biased—as more genomes and more protein features are added to an analysis.

In this study, we used an integrated probabilistic modeling approach to assess genomic correlates of protein evolutionary rate for 5,537 proteins in the yeast genome (94.5% coverage relative to the 5,861 total yeast ORFs). We assembled a list of diverse protein sequence, physicochemical, and functional genomic features with high coverage of the proteins in *S. cerevisiae* and assessed their correlations with an approximate, high coverage measure of protein evolutionary rate. To manage potential outliers, noise, and non-linear relationships, we employed robust measures of correlation, such as rank correlation and mutual information. By considering many protein attributes simultaneously, it was possible to rank them according to their degrees of association with evolutionary rate. Our high-coverage framework allows us to re-assess known genomic correlates of evolutionary rate, while simultaneously identifying new, statistically significant correlates.

In addition, we employed a logistic regression framework on binned data to assess the information contribution of sets of features in the task of predicting slowly evolving proteins. Our framework is flexible and robust, and is able to account for intercorrelation, non-linearity, and heterogeneity within features. Using this framework, we were able to group overlapping and interrelated features into natural ensembles (“meta-features”), and quantitatively assess their combined predictive power. Next, natural ensembles were evaluated in progressively larger groups to measure the independent significance of their contributions. Finally, we show that our optimal predictions of *S. cerevisiae* protein evolutionary rate are comparable to those based on paired species comparisons, and approaching those based on the lineage-independent component of evolutionary rate.

## Results

### An Approximate, High Coverage Measure of Evolutionary Rate

**Figure 1:**
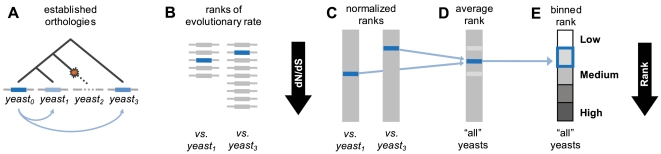
Calculating evolutionary rate. (A) We first performed conventional evolutionary rate calculation via sequence comparison between *S. cerevisiae* proteins and their annotated orthologs in five other yeasts (3 yeasts, 2 with orthologs, are depicted here for simplicity). (B) Proteins were ranked according to evolutionary rate within each paired species comparison. (C) Ranks were then normalized to account for differences in the number of orthology relationships between species. (D) A protein's normalized ranks were then averaged across all paired comparisons in which an ortholog was present. (E) Finally, average ranks of evolutionary rate were divided into five equally populated bins.

We employed an approximate, high coverage method for calculating yeast protein evolutionary rate based on multiple paired species comparisons. Figure 1 illustrates this procedure, which we outline here briefly (see the Methods section for further details). We selected five closely related yeasts for evolutionary comparison. 5,537 proteins in *S. cerevisiae* possessed an annotated ortholog in at least one of these species. Evolutionary rates were calculated for pairs of orthologous sequences following previously established procedures (e.g., codon alignment followed by dN/dS calculation). These rates were then ranked and normalized within a given paired species comparison. The evolutionary rate of a given protein is the average of its ranked, normalized rates across all paired species comparisons in which an ortholog was present.

We operate under the initial assumption that the ranked evolutionary rate of a protein is constant over time, and should therefore be approximately equal when estimated using different yeast species pairs. Averaging over multiple paired species comparisons involving *S. cerevisiae* serves to enhance the signal-to-noise ratio for evolutionary rate along the *S. cerevisiae* lineage. As our measure of evolutionary rate is approximate, we rely on relative rankings and binning to limit the influence of error. Our rankings were tested in comparison with the well established Wall et al. dataset of yeast dN/dS values [12]. Where the datasets overlap, the correlation between our ranks of evolutionary rate and those inferred from Wall et al. is high at 0.938 (i.e., 88.0% of the variation in the Wall et al. rankings can be explained by our approximate method). The advantage of our method is a substantial increase in genome coverage: Wall et al. assigned evolutionary rates to 3,038 proteins (51.8% genome coverage), while we assign ranks to 5,537 proteins (94.5% genome coverage).

### Ranking Genomic Correlates of Protein Evolutionary Rate

We collected a list of 42 high coverage protein sequence, structure, and functional genomic attributes that potentially correlate with evolutionary rate (Table 1). We ranked these features according to their absolute rank correlation coefficients with evolutionary rate. The top twenty correlates are listed in Table 2. Three categorical variables (GO slim biological process, molecular function, and cellular compartment) were excluded from this analysis as correlation coefficients cannot be computed for categorical variables. The most dominant genomic correlates of evolutionary rate are those associated with protein abundance (e.g., codon bias and absolute mRNA expression, both correlating negatively) and a subset of amino acid composition (serine and asparagine content correlating positively, and glycine, alanine, and valine content correlating negatively). Other significant correlates include: native disorder, GC content, number of interactors, degree of gene duplication, essentiality, and—reported here for the first time—number of transcriptional regulators.

**Table 1:** Protein attributes tested for potential correlation with evolutionary rate.

Meta-features	Features	# of Bins*
Amino Acid Composition	Amino Acid Content (20 total attributes)	5
Structure (Physicochemical Properties)	Predicted helix content	5
	Predicted sheet content	5
	Predicted coil content	5
	Predicted native disorder	5
	Predicted transmembrane helix content	4
	Charge (pI)	5
	Hydrophobicity (Kyte-Doolittle)	5
	Aromaticity	5
	Size	5
Function	Biological process (GO slim)	33†
	Molecular function (GO slim)	22†
	Cellular compartment (GO slim)	24†
Abundance	Absolute mRNA expression	5
	Protein expression	5
	Codon Adaptation Index (CAI)	5
	Codon bias	5
Phenotype	Essentiality	2
	Marginal essentiality	5
Network	Number of interactors	5
	Number of transcriptional regulators	5
Genome	Degree of gene duplication	4
	GC content	5

*: Continuous variables were made discrete by binning. The protein attribute we are trying to predict, evolutionary rate, was divided into 5 bins.

**†:** Number of categories within the categorical feature.

**Table 2:** Top twenty protein features ranked by absolute rank correlation with evolutionary rate.

Feature Description	Rank Correlation with Evolutionary Rate
Codon bias	−0.578
Codon adaptation index	−0.557
Protein expression	−0.486
Absolute mRNA expression	−0.467
Gly content	−0.401
Ala content	−0.390
Ser content	0.366
Asn content	0.317
Val content	−0.293
Native disorder	0.251
GC content	−0.242
Degree of gene duplication	−0.206
Sheet content	−0.191
Number of interactors	−0.160
Essentiality	−0.147
Marginal essentiality	−0.146
# of transcriptional regulators	−0.142
Hydrophobicity	−0.141
Leu content	0.105
Gln content	0.081

In addition, to deal with categorical variables and potential non-linear relationships between genomic features and evolutionary rate, we converted continuous variables into discrete variables through binning and then ranked genomic features according to their mutual information with evolutionary rate. The top twenty correlates under this scheme are listed in Table 3. The resulting order is similar to that produced by the rank correlation analysis, except that broad functional assignment (GO slim molecular function, biological process, and cellular compartment) joins protein abundance and amino acid composition as a dominant genomic correlate of evolutionary rate.

**Table 3:** Top twenty protein features ranked by mutual information with evolutionary rate.

Feature Description	Mutual Information with Evolutionary Rate
Codon bias	0.285
Codon adaptation index	0.261
Protein expression	0.189
Absolute mRNA expression	0.183
GO Slim Biological Process	0.173
GO Slim Molecular Function	0.164
Ala content	0.126
Gly content	0.115
GO Slim Cellular Component	0.109
Ser content	0.101
Asn content	0.086
Val content	0.066
GC content	0.058
Degree of gene duplication	0.055
Native disorder	0.055
Sheet content	0.040
Turn content	0.034
Essentiality	0.032
Hydrophobicity	0.030
Leu content	0.026

Statistical significance was determined from the distribution of correlation measures resulting from 100 randomizations of the feature data annotations. All correlations discussed here and listed in Tables 2 and 3 are highly statistically significant (rank correlation *z*-scores>6, mutual information *z*-scores>40; all *p*-values≪0.001).

### Genomic Correlates of Slowly Evolving Proteins

**Figure 2:**
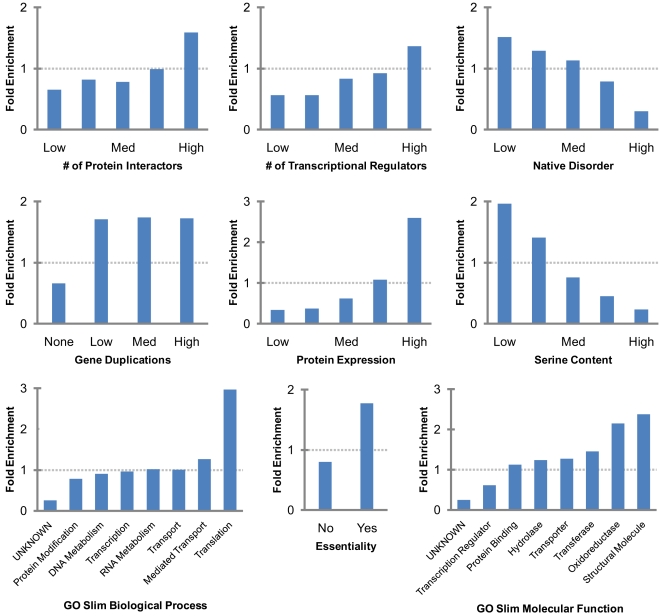
Fold enrichment plots for slowly evolving proteins (selected features). For each categorical value of a given genomic feature, we computed the fold enrichment for slowly evolving proteins, i.e. the frequency at which it occurs for the slowest evolving 20% of proteins, divided by the frequency at which it occurs over all proteins. For biological process and molecular function, only the eight most populated categories are shown. All correlations are statistically significant. Dotted lines represent the random expectation (fold enrichment = 1).

We selected the slowest evolving 20% of the proteins and asked which features best distinguish them from the remainder of the genome using a fold enrichment analysis (Figure 2). In agreement with previous studies [4]–[6], [13], [18]–[22], we found that slowly evolving proteins tend to be more abundant, be essential, have many gene duplications, and have more interaction partners. In addition, slowly evolving proteins are overrepresented in certain biological processes (such as translation) and depleted in others (such as protein modification). Similar arguments can be made for molecular function: slowly evolving proteins are common among structural molecules, but rare among transcriptional regulators. Slowly evolving proteins also tend to have low predicted native disorder [23], and have characteristic amino acid compositions [24]. We again observe a new, significant correlation between evolutionary rate and transcriptional regulation: slowly evolving proteins tend to have more transcriptional regulators. The correlation between protein evolutionary rate and number of predicted transmembrane helices is low, as are the correlations with predicted secondary structure features (not depicted). This may be in keeping with recent findings at the interface of protein structure and evolution: while structural characteristics impose clear constraints at the residue level, these constraints do not always scale to the level of whole proteins in a straightforward manner [14],[25].

### Logistic Regression as a Tool to Analyze Feature Correlation

The methods described so far have helped us to overcome some of the difficulties inherent to analyzing the relationships between protein feature data and evolutionary rate. We have been able to rank the importance of the various features in a robust statistical framework with full genome coverage, while compensating for non-linear relationships, mixed data types, and to some extent noise. We next sought to address two additional issues—joint information contribution and contribution independence—without losing the gains that our approach had already made. In order to accomplish this, we applied a logistic regression model to study the information contribution of features (and sets of features) in the task of predicting slowly evolving proteins. Logistic regression has been used in the past for predicting protein-protein interactions [26],[27]. It is capable of integrating discrete and continuous data to model non-linear relationships (through binning), and is robust against redundancy among features. This last advantage makes logistic regression particularly powerful for simultaneously modeling groups of features, a prerequisite for our next objectives.

We constructed a positive dataset consisting of the slowest evolving 20% of the proteins, and a negative dataset of the same size consisting of a random sampling of the remaining 80% of the proteins. The positive and negative datasets were then divided into five partitions. Using five-fold cross validation, we trained a logistic regression classifier using four of the five partitions, and then evaluated our model using the remaining partition as a test set. Results take the form of *correct classification rates*—i.e., when evaluating the model using the test data, the percentage of the proteins that were correctly assigned to their respective classes (slowly evolving versus *not* slowly evolving). Since all datasets were balanced prior to training and testing, a random classifier would produce correct predictions 50% of the time. This is a lower bound to which the feature-based classifications can be compared. Mathematical details of the logistic regression procedure can be found in the Methods section.

**Figure 3:**
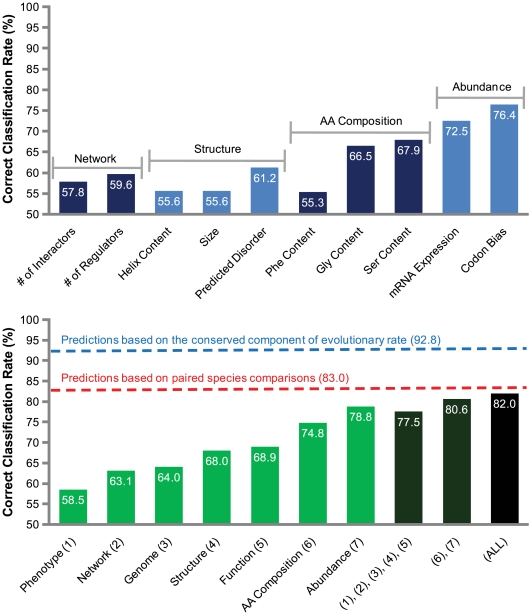
Information contribution of protein features and “meta-features” in the task of predicting slowly evolving proteins. The top frame shows the information contribution of several individual features. The bottom frame shows the information contribution of meta-features and groups of meta-features (as defined in Table 1). The blue dotted line represents predictions of *S. cerevisiae* protein evolutionary rate made using the conserved component of evolutionary rate across all yeasts. The red dotted line represents predictions based on paired species comparisons (*S. cerevisiae* versus another single yeast species, averaged over five comparisons).

The top panel of Figure 3 reports the correct classification rates for a sampling of single protein features. As with the previous methods, we are able to rank features according to the strength of their relationships with evolutionary rate. Note that while some features are closely related (under the umbrella of “amino acid composition,” for example), they may differ in their degrees of predictive power. We now turn to the major advantage of the logistic regression approach: the ability to consider sets of features simultaneously.

### Assessing Information Contribution of Feature Ensembles

In some cases, the protein features that we consider serve as proxies to some well-defined (but difficult-to-measure) property of a protein. If there are multiple proxies for a general protein property, then we expect them to be highly redundant. Other features are important in their own right, but are more tractable when considered together (e.g., amino acid composition). Features like these may also possess hidden interdependencies that we would like to model. We address these issues by grouping related features into natural ensembles, which we call meta-features. The logistic regression classifier can be trained and tested based on a meta-feature in order to assess the joint information contribution of its constituent features. Working with meta-features has several advantages: (i) it compensates for redundancy and interrelations among features, (ii) it averages out noise present in individual features, and (iii) it summarizes the many individual features into a handful of highly relevant general protein properties. Note that in all analyses based on subsets of features (including meta-feature analyses), we rely on prior expert knowledge to form the subsets, as there are too many possible subsets to exhaustively enumerate.

We pooled our 42 individual features into seven natural meta-features and then evaluated the information contribution of each using the logistic regression model (see Figure 3, bottom panel, light green bars). The meta-feature groupings are detailed in Table 1. The phenotype meta-feature is the poorest predictor of slowly evolving proteins, producing correct classifications only 58.5% of the time; this is likely due in part to the difficulty and noise associated with measuring phenotypic information. The network meta-feature provides a reasonably improved 63.1% correct classification rate; it too is likely to suffer from experimental noise. Genomic properties, structural properties, and functional annotations yield progressively improved rates of 64.0%, 68.0%, and 68.9%, respectively. Abundance, previously implicated as the single dominant factor in determining a protein's evolutionary rate [13], produces the best correct classification rate of any single meta-feature, 78.8%. Amino acid composition falls in second place with 74.8% correct classifications. Note how the individual features of the top panel compare to their related meta-features below. The meta-feature scores are always better than those of their constituent features. Some meta-features, such as abundance, have only one dominant dimension. In these cases, the component features make similar and largely overlapping contributions to predictive power, and the integration produces a minor increase in correct classification rate mainly due to noise reduction. Other meta-features, such as structure, have multiple intrinsic dimensions. In these cases, the component features make partially independent contributions to predictive power, and the integration produces a larger increase in correct classification rate.

Meta-features can be further grouped in order to test the independence of their contributions (see Figure 3, bottom panel, dark bars). When amino acid composition and abundance are grouped, we achieve a slight gain in predictive power relative to the individual meta-features (80.6% correct classifications). One explanation is that the meta-features are highly correlated, and combining them boosts predictive power through noise reduction. An alternative explanation is that each meta-feature makes a partially independent contribution to evolutionary rate prediction. As the meta-features are already noise-reduced from the combination of individual features, we conclude that independent contributions are at least partially responsible (see Figure S1 for further support). A much larger gain is made when the other five meta-features are combined (77.5%, up 8.6% compared to function alone). The combination of all seven meta-features produces further improvement (82.0%), suggesting that abundance and amino acid composition are the dominant predictors, and that other meta-features make small, individual contributions.

### Probing the Limits of Feature-Based Prediction of Evolutionary Rate

Our best feature-based predictions of slowly evolving proteins reach 82.0% correct classification rate, which is slightly beyond the midpoint of random (50%) and perfect (100%) classification. Here, we evaluate the significance of this performance in comparison with predictions based on other methods of estimating evolutionary rate.

Paired species comparison is a traditional method for estimating evolutionary rate that requires minimal genomic information. We used ranked evolutionary rates derived from a single paired species comparison (*S. cerevisiae* versus one of the other five yeasts) to predict the slowest evolving 20% of the proteins among the average rankings of the four remaining paired species comparisons (see Figure 1 and the Methods section for details of the general ranking procedure used in all analyses). This procedure was repeated five times, once for each isolated paired species comparison. On average, paired species comparison correctly identified slowly evolving proteins 83.0% of the time, which is strikingly similar to our optimal feature-based predictions. This can be interpreted either as a testament to the power of our feature-based predictions, or as a warning regarding the limitations of paired species comparison for evolutionary rate estimation.

The suboptimal performance of paired species comparison in the task of predicting slowly evolving proteins points to the existence of considerable rate heterogeneity among yeasts. In general, protein evolutionary rate can be decomposed into two components: a conserved component that is common to all yeast species, and a lineage-specific component that is unique to a particular yeast species (reflecting common and lineage-specific selection pressures, respectively). The magnitude of the conserved component of protein evolutionary rate is an important quantity, as it defines the upper limit for evolutionary rate prediction using only broad, lineage-insensitive genomic features. The intuition here is simple: genomic features that do not vary across lineages cannot distinguish the fine details of lineage-specific evolutionary rate variation. We directly estimated the conserved component of evolutionary rate that is common to all yeasts by averaging over all paired species comparisons that do not involve *S. cerevisiae*. The predictive power of this common component in the classification of slowly evolving *S. cerevisiae* proteins is reasonably high, producing 92.8% correct classifications (Figure 3); this is also the upper limit for correct classification based on lineage-insensitive (meta-)features. Our optimal feature-based predictions are able to explain three quarters of this upper limit.

The value of 92.8% is the predictive upper limit only when the integration is restricted to lineage-insensitive genomic features. How conserved are the genomic features that we consider here? Gross structural properties and broad functional assignments are likely to be conserved for homologous proteins [28],[29]. This makes biological sense: although subtle details may change in recent evolution, an all-alpha helix enzyme in the cytosol of one yeast is unlikely to become an all-beta sheet transcription factor in the nucleus of a second yeast. As a result, such features cannot predict lineage-specific evolutionary rate variation, and their predictive power is therefore bounded by the upper limit. For amino acid composition, we assessed conservation using the orthology mappings from our evolutionary rate calculation: for each yeast protein, we calculated the average ranked amino acid composition across its orthologs in the other yeast species, and correlated this average with the ranked amino acid composition in *S. cerevisiae*. The average correlation coefficient is high at 0.917, suggesting that amino acid composition is generally well conserved among yeasts, yet still subject to some degree of lineage-specific variation. As for abundance, experimental expression data for other yeast species are limited, condition-specific, and susceptible to noise. Here, we use codon bias as a proxy for expression level, and compare *S. cerevisiae*-specific values to values averaged over the other yeast species. Here the correlation coefficient is high at 0.886, again indicative of general conservation with elements of lineage-specific variation. The genomic features most likely to be variable among yeasts are network-based features, since transcriptional regulation and protein-protein interaction are known to vary between yeasts [30],[31]. The predictive limit of lineage-insensitive features is always bounded by 92.8%; as a feature's lineage specificity increases, its predictive limit can in principle approach 100%.

## Discussion

### Why Predict Evolutionary Rate?

This study focused on identifying genomic features which contribute to the task of predicting evolutionary rate. While the purpose and relevance of many prediction tasks is immediately clear—for example, predicting gene essentiality in order to avoid the difficulty and expense of experimental determination [32]—one may question the need for predicting evolutionary rate. Simple methods for evaluating evolutionary rate based on species comparisons exist (e.g., the dN/dS ratio) and can be evaluated with relative ease at the genomic scale. In the absence of such comparisons, we would have few means by which to test the validity of our predictions, given the timescale over which natural evolution operates. Why then do we wish to predict evolutionary rate?

The answer is that we are not interested so much in the predictions themselves, but rather the features which provide them. Biologists have long been interested in understanding the forces that drive evolution at various scales of life. However, our knowledge of the causal forces which underlie evolution at the molecular scale remains limited. By ranking the degree of correlation between various protein features and evolutionary rate, we hope to highlight those features which best dictate the selective constraint on a given protein. From the careful dissection of these individual correlations, one stands to gain a deeper understanding of their underlying biological significance. For example, the observed correlation between protein abundance and evolutionary rate led to biological insights regarding the evolution of translational robustness [33]. In a similar spirit, the correlation between evolutionary rate and number of transcriptional regulators that we discovered here leads to biological insights regarding the evolution of transcriptional regulation: target hubs in the transcriptional regulatory network are evolutionarily more constrained than non-hubs.

### Dominant Predictors of Protein Evolutionary Rate

Protein abundance, biological function, and amino acid composition consistently appeared in our analyses as dominant correlates of evolutionary rate. As we have mentioned before, the significance (if not dominance) of abundance is generally accepted; the significance of function has also been previously described [34]. However, the significance of amino acid composition in determining evolutionary rate has been a subject of some debate [24],[35]. The information contribution analysis indicated that the predictive power of amino acid composition is high (relative to other meta-features) in the task of classifying slowly evolving proteins. We are able to partially explain the correlation between amino acid composition and evolutionary rate by revealing a hidden correlation with protein expression (see Table 4). For example, the top three amino acids that are negatively correlated with evolutionary rate, glycine, alanine, and valine, are also the most enriched in highly expressed proteins (perhaps reflecting a preference for metabolically inexpensive building blocks). On the other hand, the correct classification rate improved when we combined abundance and amino acid composition, suggesting that amino acid composition makes at least a partially independent contribution. This additional contribution can be partially attributed to differences in amino acid mutability, as defined by Jones et al. [36] (see Table 4). For example, the top two amino acids that are positively correlated with evolutionary rate, serine and asparagine, are among the top three in terms of mutability. It is interesting to note that something as simple as amino acid composition can be highly predictive for both protein abundance and evolutionary rate.

**Table 4:** Genomic properties of the twenty amino acids.

Amino Acid	Genomic Frequency	Relative Mutability by Jones et al.	Rank Corr. w/Evolutionary Rate	Rank Corr. w/Protein Expression
Ala	5.5% (10)	0.815 (5)	−0.390 (19)	0.365 (1)
Arg	4.4% (12)	0.630 (11)	−0.048 (16)	−0.092 (15)
Asn	6.2% (6)	0.859 (3)	0.317 (2)	−0.266 (19)
Asp	5.9% (7)	0.663 (9)	−0.026 (14)	0.041 (5)
Cys	1.3% (19)	0.207 (19)	0.060 (6)	−0.106 (17)
Gln	4.0% (15)	0.641 (10)	0.081 (4)	−0.078 (13)
Glu	6.6% (4)	0.565 (12)	0.008 (10)	0.097 (4)
Gly	5.0% (11)	0.272 (17)	−0.401 (20)	0.251 (3)
His	2.2% (17)	0.717 (8)	0.044 (7)	−0.113 (18)
Ile	6.5% (5)	0.848 (4)	0.030 (8)	−0.055 (11)
Leu	9.5% (1)	0.315 (15)	0.105 (3)	−0.092 (16)
Lys	7.3% (3)	0.511 (13)	0.001 (11)	0.038 (6)
Met	2.1% (18)	0.739 (7)	−0.068 (17)	−0.084 (14)
Phe	4.4% (13)	0.283 (16)	0.010 (9)	−0.050 (9)
Pro	4.4% (14)	0.359 (14)	−0.028 (15)	−0.069 (12)
Ser	9.0% (2)	1.000 (1)	0.366 (1)	−0.295 (20)
Thr	5.9% (8)	0.891 (2)	0.066 (5)	−0.051 (10)
Trp	1.0% (20)	0.000 (20)	−0.010 (13)	−0.035 (8)
Tyr	3.4% (16)	0.272 (18)	−0.002 (12)	−0.029 (7)
Val	5.6% (9)	0.793 (6)	−0.293 (18)	0.272 (2)

Relative rankings are shown in parentheses.

### Dissecting Correlations between Protein Features

**Figure 4:**
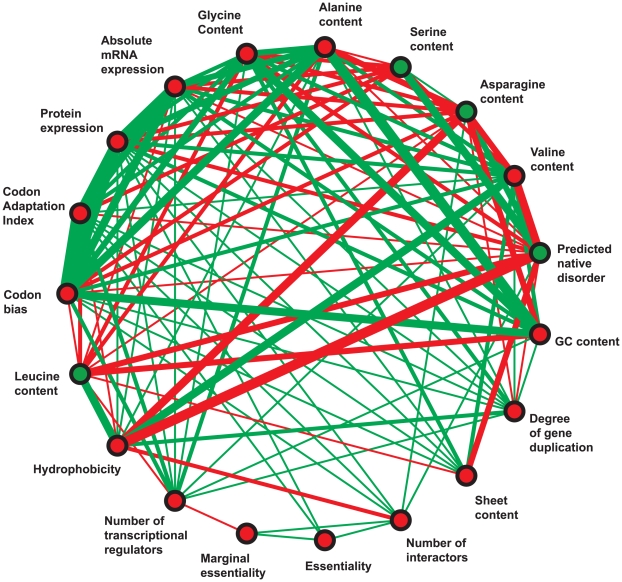
The network of correlations among top correlates of evolutionary rate. Genomic features are represented by nodes; node color corresponds to the sign of the feature's correlation with evolutionary rate (green = positive, red = negative). Edges between nodes represent a highly significant rank correlation coefficient between the two corresponding features (*r_s_*>0.1). Edge thickness corresponds to the magnitude of the correlation coefficient; edge color corresponds to the sign of the correlation coefficient (green = positive, red = negative).

The previous section highlighted the importance of understanding within-feature correlation, specifically that between protein abundance and amino acid composition, in the search for determinants of evolutionary rate. Figure 4 explores the network of within-feature correlations for the twenty numerical features that best correlate with evolutionary rate, as listed in Table 2 (glutamine content, the weakest of these correlates, has no strong within-feature correlations and is not depicted). We notice that related features can occur in tightly correlated clusters (for example, the cluster of Codon Adaption Index, codon bias, protein expression, and absolute mRNA expression). This observation reinforces the value of considering such features together as meta-features, as we have done here. In general, the network exhibits clique-like behavior, characterized by dense connections among the feature nodes. This is not entirely surprising, as many features are known to be related to one another, and they all share a common correlation with evolutionary rate. However, this rampant intercorrelation is a significant hindrance to the task of isolating specific features as evolutionary determinants using traditional multivariate statistical techniques. By taking an integrated probabilistic approach (considering all features together), we have been able to largely circumvent this issue.

Figure 4 further reveals that the vast majority of correlations between pairs of features and evolutionary rate are transitive: if features A and B both positively (or both negatively) correlate with feature C, then feature A usually correlates positively with feature B. For example, increased GC content and increased codon bias are both associated with decreased evolutionary rates. At the same time, GC content and codon bias are positively correlated with one another. These transitive correlations are easy to understand. Interestingly, we also observed non-transitive correlations, for example between evolutionary rate, number of transcriptional regulators, and marginal essentiality. Both number of transcriptional regulators and marginal essentiality are negative correlates of evolutionary rate (*r_s_* = −0.142 and −0.146, respectively; *p*≪0.001 in both cases). However, as previously noted [37], number of transcriptional regulators and marginal essentiality correlate in a negative manner with one another (*r_s_* = −0.104, *p*≪0.001). The observed non-transitive correlations are statistically significant (*p*≪0.001), although we note that the correlations are rather weak and account for 1.1% to 2.1% of the variance. This seemingly counter-intuitive observation can be explained in the following way. Slowly evolving proteins can be divided into two largely non-overlapping groups: (i) those that are important under all conditions, meaning that they are essential, but not necessarily highly regulated, and (ii) those that are important only under specific conditions, which may experience sophisticated regulation, but are not necessarily annotated as essential. Proteins in the first group drive the negative correlation between essentiality and evolutionary rate, while proteins in the second group drive the negative correlation between number of regulators and evolutionary rate. This explains the observed non-transitive correlations among evolutionary rate, number of transcriptional regulators, and marginal essentiality.

The patterns of correlation among features in Figure 4 provide further insights into our observed correlation between evolutionary rate and number of transcriptional regulators. Number of regulators is correlated with many other genomic features, most significantly with codon bias and Codon Adaption Index. Highly regulated proteins, though not necessarily essential or even expressed under laboratory conditions, may be strongly selected in the real world for their roles in stress response. Take, for example, HSP26—a player in the yeast response to heat shock, and the most highly regulated protein in our dataset. This protein is neither expressed nor essential under laboratory conditions. However, its coding sequence contains high codon bias, consistent with selection for efficient translation under stress. We therefore expect that translational selection and selection for the protein's stress-induced function have constrained its evolution in the wild.

### Strengths and Limitations of the Methodology

The integrated probabilistic approach we have taken in this study has both drawbacks and advantages. Like other correlational approaches, our approach is not able to distinguish correlation from causation, nor is it able to isolate cause from effect. We do not explicitly model the noise within the feature data as some other methods do [15], which will tend to underestimate the predictive power of single features. On the other hand, the effect of noise is minimized by the binning of single features and introduction of meta-features. Our approach is flexible and robust, and is able to distinguish between dominant correlations and marginal ones. We are able to consider any features we choose, including those that are categorical (rather than continuous) or correlated with evolution in a non-linear manner. Furthermore, our approach compensates for redundancy among features, which, as with noise, we expect to be significant. Most importantly, our analyses feature high coverage of the yeast genome, thus making our results highly general. Accomplishing this requires the introduction of several approximations (a relaxed definition of evolutionary rate, collecting feature data from a single species, and modeling missing data), though none of these are found to have a major effect on accuracy.

### Closing Remarks

To our surprise, we found that integrating a diverse collection of single-genome features was roughly equivalent to paired species comparison for identifying slowly evolving proteins, but still worse than what lineage-insensitive features can in principle predict. Our conclusion from this finding is that the dominant, independent correlates of evolutionary rate are likely known, even though other significant and interesting correlates may remain to be found (see [38] for one recent example). Further dissection of individual correlations between protein features and evolutionary rate will be needed in order to gain a deeper understanding of their biological significance. As we have demonstrated in the cases of amino acid composition, protein abundance, essentiality, and number of transcriptional regulators, there is also great insight to be had by exploring the relationships between protein features.

## Methods

### Calculating Protein Evolutionary Rate in Yeast

We based our measure of protein evolutionary rate on comparisons between *Saccharomyces cerevisiae* and five related yeast species: *S. paradoxus*, *S. mikatae*, *S. bayanus*, *S. castellii*, and *S. kluyveri*. Of the 5,861 open reading frames (ORFs) in the *S. cerevisiae* genome, 324 had no annotated orthologs [39] among these species, and were therefore discarded. The remaining 5,537 ORFs (94.5% genome coverage) each have at least one ortholog [39] in at least one of the five related yeasts; this group forms the basis of our evolutionary rates dataset. We first performed local alignment [40] between each ORF and its annotated orthologs across the five species. If an ORF had multiple orthologs in a given species, only the most significant alignment with the highest score was saved. These protein alignments (having 95% ORF coverage, on average) were used to generate corresponding DNA codon alignments, which were then piped into PAML [41] to calculate dN/dS [42]. All dN/dS values resulting from a given paired species comparison (i.e., *S. cerevisiae* versus one other yeast) were then treated as follows: (i) dN/dS was first adjusted according to the method of [43] to compensate for selection at synonymous sites; (ii) adjusted dN/dS values were next sorted and converted to ranks; and (3) ranks were normalized relative to the total number of alignments considered in the paired species comparison. Finally, a single evolutionary rate was generated for a given ORF by averaging over its normalized ranks from all paired species comparisons in which an ortholog was present and dN/dS was successfully calculated. The values were then re-ranked and divided into five equally populated bins corresponding to *low*, *medium low*, *medium*, *medium high*, and *high* evolutionary rate. This procedure is summarized in Figure 1. We provide the average ranks and bins of yeast protein evolutionary rate in Table S1. Sequence data for *S. kluyveri* were obtained from [44]; all other sequence data were obtained from [45].

### Collecting Protein Features

Basic protein information about each ORF was downloaded from the *Saccharomyces* Genome Database [45]. Protein GO annotations were downloaded from the Gene Ontology project website [46]. Protein-protein interaction data were downloaded from BioGRID [47]. Transcriptional regulatory data were obtained as described in (Wang, Zhang, and Xia, submitted). Protein native disorder was predicted from sequence using DISOPRED [48]. Transmembrane helix content was predicted from sequence using TMHMM [49]. All other feature data were assembled following the procedures outlined previously [50]. Note that the majority of our features are derived or predicted from sequence alone, and therefore have high coverage of the yeast genome. At the same time, some features that we considered contain missing data. In the mutual information and subsequent analyses, missing data are treated as a separate feature bin. For example, the mRNA expression feature now has six categorical values: *high*, *medium high*, *medium*, *medium low*, *low*, and *missing*. These “completed” features are then correlated with evolutionary rate. Here, we assume that missing data bins such as “unknown biological process” or “missing mRNA expression” can be correlated with evolutionary rate just as we would correlate regular feature bins, such as “constituent of the ribosome,” or “high mRNA expression level.” This approach involves fewer assumptions about the nature of missing data than alternative strategies, such as listwise deletion, mean substitution, and imputation.

### Rank Correlation Coefficient and Mutual Information

Given *N* pairs of quantities (*x_i_*, *y_i_*), *i = 1*,*…*,*N*, the Spearman rank correlation coefficient *r_s_* is computed in the following way:
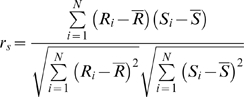
where *R_i_* is the rank of *x_i_* among the other *x*'s, *S_i_* is the rank of *y_i_* among the other *y*'s.

The mutual information *I* between two discrete random variables *X* and *Y* is computed in the following way:
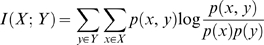



### Logistic Regression Classifier

For a given protein, we want to predict the class label *y_i_* (1 if the protein evolves slowly, and 0 otherwise) by integrating genomic features *F*. There are *m* categorical features, *F_1_*, …, *F_m_*, where each feature *F_j_* can take on *r_j_* different values, *f_j1_*, *f_j2_*, …, 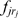. The training set, {(*F^(i)^*, *y^(i)^*); *i = 1,…,n*}, contains *n* samples. Logistic regression can be expressed as the following weighted voting scheme:
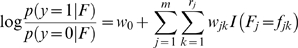
Where *I* is the indicator function—*I(X)* is 1 when statement *X* is true, and 0 otherwise. *w_jk_* are weights associated with each piece of evidence. *p*(*y = 1|F*) is the probability that the protein evolves slowly given the features. The protein is predicted to evolve slowly if and only if *p*(*y = 1|F*) is larger than 0.5.

All weights are chosen to optimize the following log-likelihood function for the training set, i.e. the log-probability of observing the data given the weights:
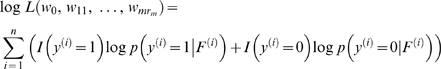
The right-hand side of the above equation measures the agreement between the actual class labels *y* and the predictions *p*(*y|F*).

## Supporting Information

Figure S1 Noise reduction and independent contribution during feature integration. When integrating abundance features in various meta-feature combinations, predictive power increases and gradually levels off due to noise reduction. Addition of the amino acid composition meta-feature results in a marked jump in predictive power, indicating an independent effect.
(0.42 MB PDF)

Table S1 Rankings and associated bins for yeast protein evolutionary rate.
(0.44 MB XLS)
