# Supplementary figures and images for: Integrated Assessment of Genomic Correlates of Protein Evolutionary Rate
Source: PLoS Comput Biol. 2009 Jun 12;5(6):e1000413. doi: 10.1371/journal.pcbi.1000413 (PMC2688033; doi:10.1371/journal.pcbi.1000413)

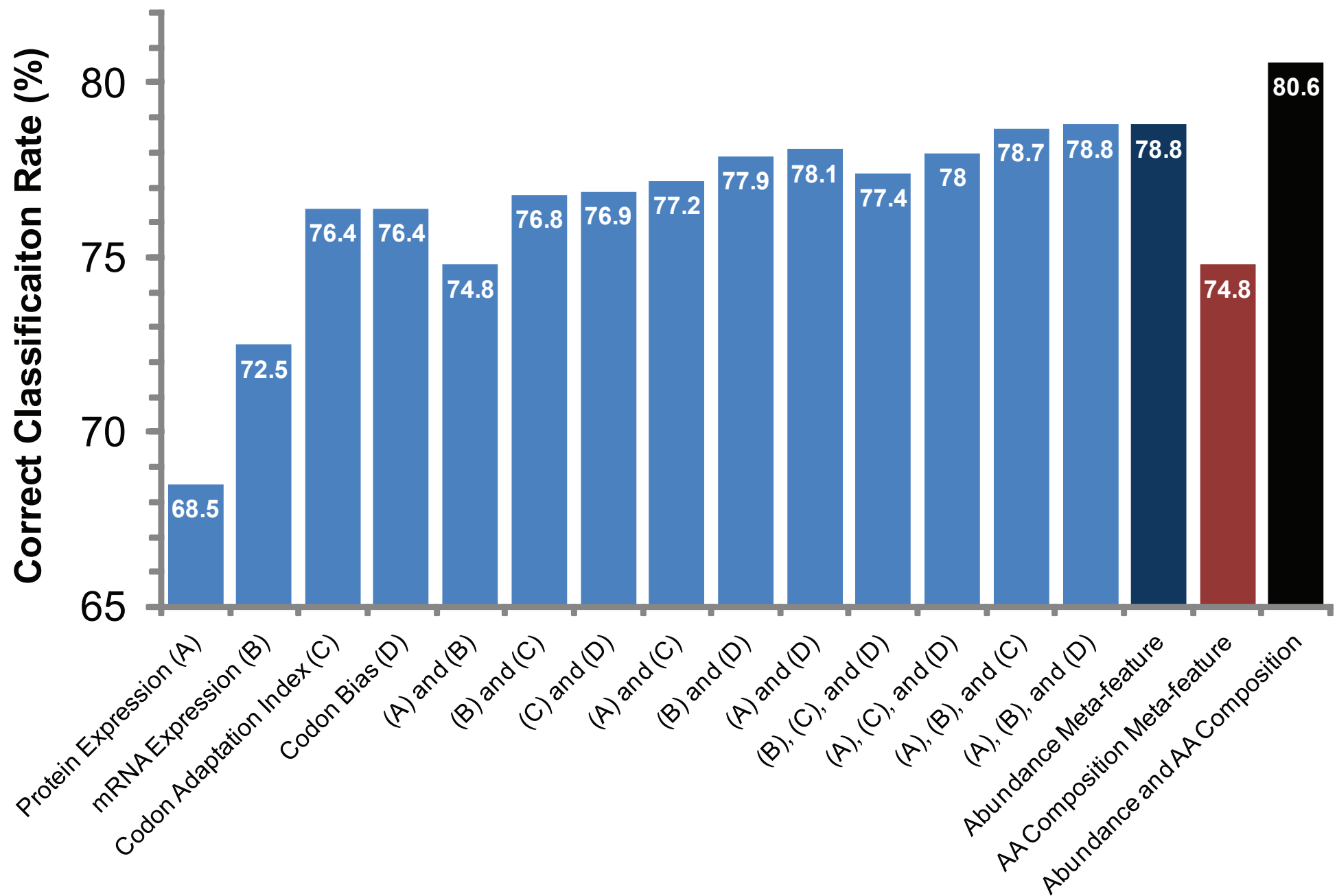

Supplement: Figure S1 — Noise reduction and independent contribution during feature integration. When integrating abundance features in various meta-feature combinations, predictive power increases and gradually levels off due to noise reduction. Addition of the amino acid composition meta-feature results in a marked jump in predictive power, indicating an independent effect. (0.42 MB PDF) [file pcbi.1000413.s001.pdf]
